# Our New York Letter

**Published:** 1892-07

**Authors:** Thomas H. Manley

**Affiliations:** Visiting Surgeon to Harlem Hosqital, New York


### Correspondence.

### OUR NEW YORK LETTER.

NEW YORK, June 18, 1892.

At a recent meeting of the New York Academy of Medicine, Dr. Paul F. Munde, professor of gynæcology at the New York Polyclinic, read a very interesting and instructive paper on the conservative treatment of salpingitis, in which he stated that he believed the time had come when those who are accustomed to perform laparotomies for the removal of diseased tubes and ovaries must beat a hasty retreat from their present plan of procedure. He is convinced that in the past many uterine appendages have been removed which, by a little patience and perseverance on the part of the physician, might have been saved. This applies in particular to cases of catarrhal salpingitis in which the patient complains of pain in one or both ovarian regions which did not yield promptly to local applications of iodine, etc., and in which the appearance, perhaps, at irregular intervals of a muco-purulent discharge from the vagina denoted the existence of a pyosalpinx. The mere presence of a catarrhal salpingitis, with or without adhesion, the mere presence of a certain amount of pain in these regions, does not of itself demand the removal of the diseased organs. A true pyosalpinx, however—that is, the presence of pus in the Fallopian tubes—calls for its evacuation. If the pus is contained in only one tube and the tube is adherent, Dr. Munde considered it justifiable to aspirate *per vaginam*, and finding pus, to enlarge the incision, wash out the tube, and in this way endeavor to produce obliteration of the calibre of the tube without exposing the patient to the risk of abdominal section. When both appendages are diseased, it is not worth while to resort to vaginal aspiration, but to remove the diseased organs by laparotomy, which is the only correct treatment to follow.

Acute inflammations of the tube are treated by rest in bed, hot vaginal douches, hot poultices, opium and antipyretics.

As the case becomes subacute, mild applications of the tincture of iodine, with equal parts of glycerine, may be made to the vault of the vagina, in connection with glycerine tampons and warm sitz baths.

Dr. Hanks agreed with the author of the paper in the principles he set forth, and said we should never perform a laparotomy for the removal of a diseased tube unless we were satisfied that the tube was never to be of any further use to the patient.

Dr. Harrison concurred in the views of Dr. Munde with regard to the too frequency of laparotomies in women. In acute cases of salpingitis, he thought there was nothing equal to the application of ice for the relief of pain and fever.

Dr. Polk approved of the methods presented by Dr. Munde, in the paper that had just been read. The results that have been secured by drainage of the uterus, justified us in believing that it will undoubtedly limit operative cases. The other methods of treatment, such as rest, heat, cold, the application of tampons, etc., are familiar to all, but this one of drainage is by far the most important, and he now employs it in all forms of acute salpingitis.

Dr. Edebohls emphasized the value of ichthyol in the treatment of pyosalpinx. He recommends its use energetically, internally as well as by inunction to the external abdominal and vaginal walls. By its employment, he claims the pain will be relieved more quickly than by massage or electricity.

At a recent meeting of the New York Surgical Society held at the Academy of Medicine, Dr. Gerster presented a woman, 31 years of age, from whom he had removed one kidney while undoubted disease of the other existed. The tumor occupied the entire left side of the abdomen and was but slightly movable. The condition of the patient was very bad. The tumor contained pus which was evacuated by the operation of nephrotomy on March 3d, 1890. Improvement followed, but as elevations of evening temperature still persisted and the discharge was profuse, it was decided to remove the kidney. Nephrectomy was accordingly done on January 31st, 1891. The pedicle was secured by an elastic ligature and the peritoneum shut off by continuous sutures. The wound was treated by the open method. The ligature came away on the 13th day and the patient made a good recovery; in four months her weight increasing from 99 to 135 pounds.

Dr. Charles McBurney has resigned the chair of surgery at the College of Physicians and Surgeons, this city, and has been succeeded by Dr. R. F. Weir, professor of clinical surgery at the same institution. The appointment of Dr. Weir has given general satisfaction to the students and faculty of the college.

Dr. W. Gill Wylie, in a recent lecture on puerperal fever at the N. Y. Polyclinic, made use of the following language: "I believe now, and I say it with perfect sincerity, that nine cases out of ten of puerperal fever that I am called upon to treat, in from twelve to twenty-four hours after the onset of the attack, I will cure by simply regulating the bowels and washing out the uterus systematically and thoroughly at frequent intervals. If you follow the instructions of the text books, you will find that they advise the washing to be done but once in eight hours. I have proved to my own satisfaction the fallacy of this advice. The germs are not destroyed by the first or the second washing and they develope anew in eight hours. If I am called to a case of puerperal fever, and within six hours or so after frequent washing out the uterus the temperature does not fall to normal, I make up my mind that the poison has entered the tissue of the patient and I at once proceed to open the belly. I generally find there an abscess present."

---

CLEMIANA CHEMICAL CO., ATLANTA, GA.:

*Gentlemen*:—Having given the preparation known as verrhus Clemiana a thorough and extended trial, on hospital and private patients, I can most cheerfully recommend it, as serving an excellent purpose in those conditions resulting from specific and scrofulous diseases. I have found it act with almost specific energy in strumous joint diseases of childhood and in many cases of chronic malarial poisoning which have resisted quinine and arsenic. I am yours very truly,

THOMAS H. MANLEY, M. D.,
Visiting Surgeon to Harlem Hosqital, New York.

New York, June 13, 1892.

